# The Consequence of Oncomorphic *TP53* Mutations in Ovarian Cancer

**DOI:** 10.3390/ijms140919257

**Published:** 2013-09-23

**Authors:** Pavla Brachova, Kristina W. Thiel, Kimberly K. Leslie

**Affiliations:** 1 Molecular and Cellular Biology Program, University of Iowa, Iowa City, IA 52242, USA; E-Mail: pavla-brachova@uiowa.edu; 2 Department of Obstetrics and Gynecology, Carver College of Medicine, University of Iowa, Iowa City, IA 52242, USA; E-Mail: kristina-thiel@uiowa.edu; 3 Holden Comprehensive Cancer Center, Carver College of Medicine, University of Iowa, Iowa City, IA 52242, USA

**Keywords:** *TP53*, oncomorphic mutation, ovarian cancer, mutant p53, chemoresistance

## Abstract

Ovarian cancer is the most lethal gynecological malignancy, with an alarmingly poor prognosis attributed to late detection and chemoresistance. Initially, most tumors respond to chemotherapy but eventually relapse due to the development of drug resistance. Currently, there are no biological markers that can be used to predict patient response to chemotherapy. However, it is clear that mutations in the tumor suppressor gene *TP53*, which occur in 96% of serous ovarian tumors, alter the core molecular pathways involved in drug response. One subtype of *TP53* mutations, widely termed *gain-of-function* (*GOF*) mutations, surprisingly converts this protein from a tumor suppressor to an oncogene. We term the resulting change an oncomorphism. In this review, we discuss particular *TP53* mutations, including known oncomorphic properties of the resulting mutant p53 proteins. For example, several different oncomorphic mutations have been reported, but each mutation acts in a distinct manner and has a different effect on tumor progression and chemoresistance. An understanding of the pathological pathways altered by each mutation is necessary in order to design appropriate drug interventions for patients suffering from this deadly disease.

## 1. Introduction

One of the main goals of clinical research is to establish better drug regimens for patients suffering from specific diseases. In the field of ovarian cancer research, the current standard therapy of platinum and paclitaxel has been in place without change for almost twenty years [1]. The lack of successful new treatments for ovarian cancers prompted a comprehensive genomic analysis through The Cancer Genome Atlas (TCGA) project, with the goal of identifying genetic abnormalities in ovarian tumors that could influence the pathophysiology of the disease and response to therapeutic drugs [2]. The most resounding finding from this study was the identification of mutations in a single gene, *TP53*, in 96% of all serous ovarian tumors. *TP53* encodes p53, a tumor suppressor that acts as a major control hub for the cellular response to various stresses, including DNA damaging chemotherapy. Once activated, p53 protects against cancer by functioning as a sequence-specific transcription factor or through protein: protein interactions, activating cell cycle arrest, apoptosis, and DNA damage repair.

**Figure 1:**
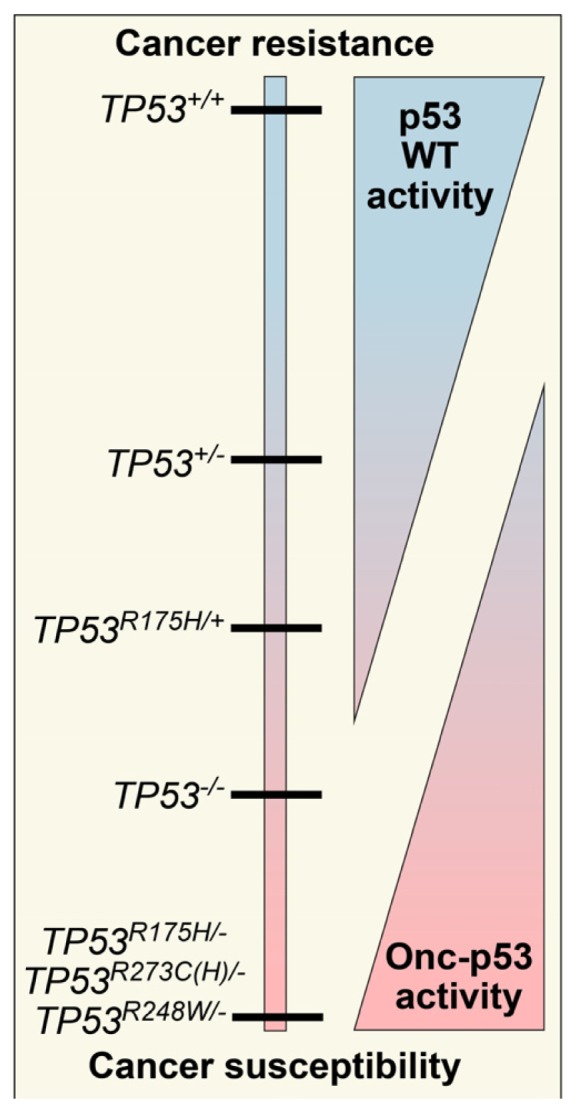
The spectrum of protection against cancer provided by WT p53. As copies of WT p53 (*TP53*^+/+^) are lost, cancer protection decreases. When oncomorphic mutations are acquired, cancer susceptibility is increased.

Unlike other tumor suppressor genes such as *RB1* or *APC* that are largely inactivated by mutations that result in deletion or truncation [3], the majority of *TP53* mutations are single base-pair substitutions that result in the hyper-stabilization of the encoded protein. Mutations are primarily localized to the highly conserved DNA binding domain and inactivate wild type (WT) p53 function. The spectrum of mutations in *TP53* is extremely diverse, and a few particular mutations can actively promote oncogenesis (Figure 1). Historically, these types of mutations have been called *gain-of-function* (*GOF*) mutations. However, the term *GOF* is arguably a misnomer because the WT function of p53 is lost while oncogenic function is gained, thereby contributing to confusion about the biology of these mutations. Thus, we propose a new term for mutations that convert a tumor suppressor into an oncogene: oncomorphism. In this review, we discuss the most common *TP53* mutations in ovarian cancers that confer oncomorphic activity.

Many clinical studies have attempted to correlate the presence of a *TP53* mutation with patient survival or the development of chemoresistance [4–22]. However, the conclusions of these studies are conflicting, due in large part to insufficient analysis and inadequate methods. First, the indiscriminate classification of all *TP53* mutations as the same may under-represent the impact of individual mutations. Second, a majority of studies rely solely on immunohistochemistry (IHC) to determine if *TP53* is mutated. IHC staining of p53 is commonly elevated when *TP53* is mutated because most missense mutations hyper-stabilize the protein [23–25], as opposed to WT p53 that is normally degraded and expressed at low levels. This method has the potential to produce a high frequency of both false negatives and false positives. Consistent with this notion, a recent meta-analysis investigated the relationship between the presence of a *TP53* mutation and clinical outcome in ovarian cancer patients following chemotherapy [24]. The authors established stringent criteria for inclusion of studies in the meta-analysis. Strikingly, only six of 64 clinical publications fulfilled the criteria. The most common reasons for exclusion were the use of IHC as the only method to identify the presence of a *TP53* mutation, sequencing only partial segments of the *TP53* gene, and importantly, bundling all mutations in the same group. Several emerging efforts acknowledge the biological differences of p53 mutant proteins when correlating *TP53* mutational status with patient outcomes. Indeed, the past 20 years have laid a significant foundation, demonstrating the function of distinct *TP53* mutants in cultured cells and animal models. It is clear that certain mutations enable p53 to acquire new, oncogenic behaviors with potential clinical significance. This review will analyze the most common oncomorphisms of p53 in ovarian cancer and the pathophysiological mechanisms contributing to cancer progression. Given that survival of patients who become chemoresistant and recur is very low, a better understanding of the biology of distinct p53 mutant proteins is vital to predict response to tumor therapies as well as to identify future platforms for novel treatment strategies based on individual *TP53* mutations.

## 2. Defining *TP53* Mutations

In order to use *TP53* mutations as biomarkers to predict patient response to chemotherapy, there needs to be a clear understanding of the biologic consequence of each mutation. We argue *TP53* mutations should be categorized based on their functional consequences: WT, loss of WT function, partial loss of function, and oncomorphic. A significant number of *TP53* mutations have been reported in the literature, though only a small proportion has been characterized experimentally. Unfortunately, *TP53* sequence alone cannot provide definitive information regarding its function in the setting of the tumor, thereby limiting the predictive value of *TP53* mutational status with regards to prognosis and response to therapy. Defining the cellular effects of *TP53* mutations requires exhaustive *in vitro* and *in vivo* studies to determine the consequence of a particular mutation. True *loss–of-function* (*LOF*) mutations, which are classically defined as loss of p53 protein expression, can be found in the form of insertions and deletions that cause a frame shift, splice mutations that cause major changes in the protein structure, or nonsense mutations that abruptly terminate translation. For these *LOF* mutants, typically p53 cannot be detected at the protein level, though some exceptions exist [26]. For example, an analysis of *TP53* mutations in bone and soft tissue sarcomas found positive p53 staining in 1/10 tumors analyzed with LOF *TP53* mutations [27].

Mutations can also occur in the form of splice mutations at the intron: exon splice junctions, resulting in alternate p53 splice isoforms with *C*-terminal truncations [28–31]. Expression of these splice variants in ovarian cancer are associated with a worse prognosis and resistance to chemotherapy [31].

Missense mutations can also result in loss of WT p53 function, with a hyper-stabilized but non-functional p53 detectable by IHC. Partial *LOF* mutations that retain some WT p53 function, but lose other functions are more difficult to predict. For example, substitution of arginine at codon 175 for proline (R175P) is a rare missense mutation, and cells with this mutation are capable of inducing cell cycle arrest but fail to activate apoptosis [32]. It is currently unknown how partial *LOF* mutations can affect patient response to chemotherapy or if these mutations confer any oncogenic function.

Oncomorphic mutations are due to missense mutations that produce p53 proteins with oncogenic functions that are entirely independent of WT p53 functions. Oncomorphic p53 activity requires the absence of the second WT *TP53* allele.

**Figure 2:**
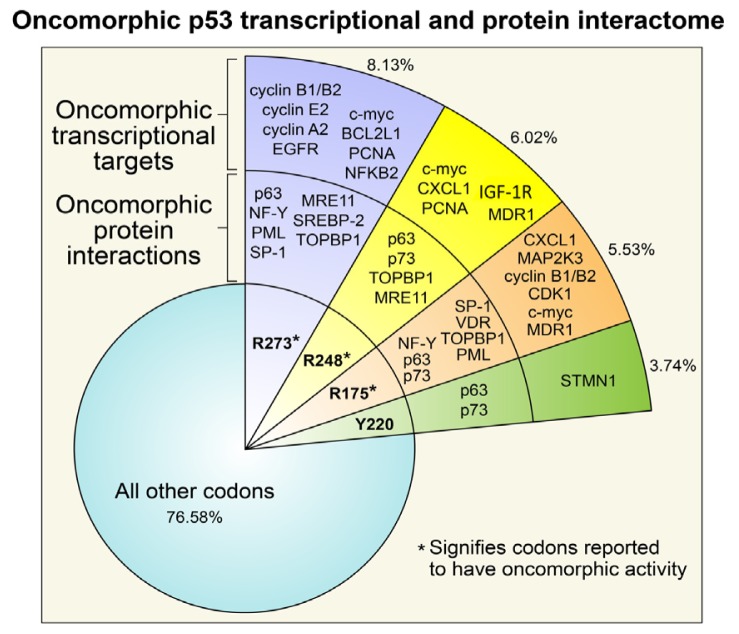
Hotspots for *TP53* mutations. Mutations that occur at a frequency greater than 3% are highlighted. Certain p53 mutants have oncomorphic activity (denoted by ^*^), functioning through novel protein interactions as well as novel transcriptional targets to promote cell survival and potentially chemoresistance. Codons in the “other” category include those that produce non-functional p53 or have not been characterized to date.

To understand the distribution of *TP53* mutations in ovarian cancer, we analyzed data available from the public database of *TP53* mutations from the International Agency for Research on Cancer using the selection criteria of “ovarian carcinoma” [33,34]. We found that approximately 15% of all *TP53* mutations are null mutations resulting in a true *LOF* (*i.e.*, nonsense or frame shift mutations). Previous analyses suggest that splice mutations occur at a low frequency of about 1%–2% [30,33]; however, our analysis of serous ovarian cancer patient data available from the TCGA revealed splice site mutations in 32/306 (10%) of patients. The majority of spice site mutations (10/32, 31%) occurred at the exon 4 boundary. Interestingly, particular codons in *TP53* are mutated at a higher frequency than others, termed “hot spot” mutations. In 615 reported ovarian cancer patient tumors, the most frequent hot spot mutations occurred at codons *R273*, *R248*, *R175*, and *Y220*, each at a frequency of more than 3% (Figure 2). The additional mutations are poorly characterized and occur at lower frequencies. It is of note that many of the hot spot mutations are associated with oncomorphic activity and/or create a highly-stable p53 protein [35–37], but their association with sensitivity to therapy in ovarian cancer is unknown. Thus, due to gained oncogenic abilities, oncomorphic p53 can be considered a renegade p53, and individual mutations in the gene must be understood before attempting to find associations between *TP53* mutations and patient outcomes.

## 3. Oncomorphic *p53* Mutations

Oncomorphic p53 proteins were first identified over two decades ago, when different *TP53* mutants were introduced into cells devoid of endogenous p53 [38,39]. Among all cancers, the most common oncomorphic mutations are at positions *R248*, *R273*, and *R175*, and in ovarian cancers the most common oncomorphic *TP53* mutations are at positions *R273*, *R248*, *R175*, and *Y220* at frequencies of 8.13%, 6.02%, 5.53%, and 3.74%, respectively [33,34]. In *in vitro* studies, cells with oncomorphic p53 demonstrate increased invasion, migration, angiogenesis, survival, and proliferation as well as resistance to chemotherapy [35,37,40,41]. Further compelling evidence for the phenomenon of oncomorphisms in p53 was shown *in vivo* using transgenic mice expressing various oncomorphic mutations. When compared with WT p53, heterozygous or knockout mice (*p53*^+/−^ or *p53*^−/−^, respectively) mice with an oncomorphic allele showed a different and more broad tumor spectrum, as well as increased metastases [35,42,43]. For example, *p53*^−/−^ mice develop many lymphomas and sarcomas [44]. Knock-in mice with the *p53 R175H* mutation also display a high incidence of lymphomas and sarcomas, but in addition have a significant number of carcinomas and an increased metastasis rate [42,45]. As another example, mice with liver-specific expression of p53 R246S displayed increased liver carcinomas [46]. An in-depth analysis of mouse models of mutant p53 proteins has been reviewed elsewhere [47,48] that focuses on the role of individual mutations in the tumorigenesis. This review will focus on the role of individual *TP53* mutations in the response to chemotherapy.

Four distinct mechanisms have been proposed to explain p53 oncomorphic activity (Figure 2) [49,50]. In the first mechanism, a particular missense mutation alters the sequence specificity required for p53 transactivation of target genes, resulting in transcription of novel targets [51–63]. Although many of these transcriptional targets of oncomorphic p53 are known, to date no consensus sequence(s) have been identified for each mutant or the oncomorphic p53 mutant family. The second mechanism is via indirect transcriptional activation of genes mediated by oncomorphic p53 binding to non-typical transcription factors or transcriptional cofactors [64]. The third mechanism also involves protein: protein interactions that influence transcription of other genes. Specifically, oncomorphic p53 binds to and sequesters other transcriptional factors or co-activators necessary for a normal stress response [57,64–72]. The fourth mechanism is through oncomorphic p53 interaction with novel proteins to either enhance or decrease their activity [73,74]. Taking all of these differing mechanisms into consideration, it is likely that the function of each oncomorph varies greatly depending on the mutation, and potentially between tumor types that harbor unique mutations in other genes in addition to an oncomorphic *TP53* mutation. Few studies have identified and explored *TP53* oncomorphic mutations in ovarian cancer cell lines or animal models of the disease, and an in-depth analysis would be particularly relevant considering the frequency of *TP53* mutations. As each p53 oncomorph has unique properties, it is of importance to conduct studies in ovarian cancer models in order to understand how these alterations affect ovarian cancer biology and the developing chemoresistance. The following sections will describe in more detail the known functions of the p53 oncomorphs most frequently detected in ovarian tumors.

### 3.1. R273 p53

Mutations in codon *R273* are the most common *TP53* alterations in ovarian carcinomas, with the most frequent mutations resulting in an amino acid change to histidine or cysteine. Mutation of *R273* significantly alters the target DNA sequence without causing structural distortions, thus maintaining the ability to bind DNA [75,76]. *In vivo* studies in mice with an engineered *R273H* mutation in the endogenous locus (*p53*^R273H/−^) demonstrate no difference in survival as compared to *p53*^−/−^ mice [35]. However, the *p53*^R273H/−^ mice develop more carcinomas, including high numbers of lung adenocarcinomas and squamous cell carcinomas. Additionally, cells cultured from these tumors demonstrate an increased capacity to proliferate [35]. In studies of lung and bladder cancer cells, overexpression of R273H p53 on a *TP53* null background results in resistance to cisplatin [40,41]. It remains unclear, however, whether R273H p53 mediates broad chemoresistance or resistance to a particular class of DNA damaging agents. For example, the same studies that observed resistance to platinum-based chemotherapy found that cells with R273H p53 retain sensitivity to the topoisomerase II poison etoposide [40].

The mechanisms underlying increased resistance to some chemotherapeutic agents likely involve new protein interactions as well as new transcriptional targets [51–53,56,57,61,77,78]. Novel protein interactions have been described between the p53 R273 mutant proteins and the transcription factors NF-Y, SP1, SREBP-2, MRE11, and p63. For example, Di Agostino *et al.* showed that after DNA damage, R273H/C p53 complexes with the transcription factor NF-Y on the promoters of the cell cycle genes cyclin A, cyclin B, cdk1, and cdc25c and activates their expression [57,79]. Gene activation of the target genes is mediated through p300, the histone acetyltransferase, and the transcriptional co-factor TopBP1 [64]. These interactions could induce progression of the cell cycle through S phase and the transition from G2 to M phase. Another transcription factor that has been shown to cooperate with R273H/C p53 is SP-1, which is necessary for full R273H/C p53 transactivation in the context of human immunodeficiency virus type 1 (HIV-1) long terminal repeat (LTR)-mediated transcription [80]. Whether the oncomorphic p53 R273 interacts with SP-1 in ovarian cancer cells is unknown, but if enhanced SP-1-mediated transcription occurs, it is likely to have an important impact on cell function.

In addition to mediating chemoresistance, interactions between R273H/C mutant p53 and other proteins may contribute to oncogenesis or tumor progression. The best characterized R273H p53 interaction is with its family member p63. Specifically, p63 activity is inhibited by R273H p53 binding, which in turn promotes TGF-β-induced metastasis [81]. The loss of p63 activity, due to a dominant negative effect of R273H p53, can also increase invasive and metastatic properties through enhanced recycling of integrins and EGFR to the plasma membrane [82]. The result of this is enhanced EGFR phosphorylation and activation of AKT pro-survival signaling. Indeed, many tumors with this particular mutation have a significant correlation between p53 expression and AKT phosphorylation [82]. The oncomorph R273H/C association with SREBP transcription factors results in a novel p53-mediated regulation of mevalonate genes, which are involved in statin and sterol biosynthesis [60]. Depletion of R273H/C p53 is sufficient to alter the morphology of breast cancer cells to a less invasive phenotype [60], which is speculated to be related to abrogation of the mevalonate pathway. Another novel protein interaction was recently identified between R273H and the nuclease MRE11 [73]. MRE11 is essential for repairing double-strand DNA breaks and is recruited to damaged DNA as part of the larger MRN DNA damage complex, consisting of Mre11, Rad50, and Nbs1. Interaction of R273H p53 with MRE11 prevents the DNA damage protein complex from being recruited, thereby promoting genomic instability and contributing to the oncogenic phenotype. Interestingly, WT p53 also interacts with several of these transcription factors (*i.e.*, NF-Y and SP-1), although the interaction has the opposite effect compared to oncomorphic p53 [58,69,83]. Thus, the net effect of the interactions of p53 R273H/C with transcription factors is to increase proliferation and survival, mechanisms that are very important in the development of chemoresistance.

### 3.2. R248 p53

The codon *R248* is the second most commonly altered amino acid in ovarian carcinomas, occurring at a frequency of 6.02% [33,34]. The most frequent amino acid change replaces the arginine with a tryptophan or glutamine (*R248W* or *R248Q*). This alteration does not significantly affect the overall conformation of the p53 protein but changes the DNA binding response element, thereby altering interactions with DNA [84]. Cellular studies indicate that mutations of codon *248* can increase oncogenicity through increased invasion and chemoresistance to particular drugs. In lung cancer cells, although there are no differences in the proliferation rate of clones stably expressing this mutant, cells with this mutation do display significantly higher migration [36]. The mechanisms behind this phenomenon remain unknown. Studies analyzing sensitivity to drugs in hepatocellular carcinoma cells found that the mutant R248Q confers chemoresistance to doxorubicin and paclitaxel. This effect is mediated by the upregulation of multidrug resistance gene 1 (MDR1, also ABCB1 or *P*-glycoprotein) [37]. As with R273H p53, lung cancer cells that overexpress the R248Q mutant show no difference in sensitivity to etoposide [40]. The differential sensitivity to chemotherapeutics is most likely due to the distinct mechanisms of action of each agent. In studies of *R248W TP53* knock in mice (*p53*^R248W/−^), expression of R248W p53 markedly accelerates the development of lymphomas and sarcomas [73]. In addition, cells cultured from the tumors demonstrate an abrogation of the G2/M checkpoint following radiation, which leads to increased genomic instability as demonstrated by the 78% rate of translocations in pre-tumor R248W thymocytes. Recently, a knock in mouse model of the *TP53 R248Q* mutant was created, and these mice display accelerated tumor onset as well as a shortened survival [85].

The p53 mutant R248 can activate the transcription of several genes associated with chemoresistance, namely c-Myc, CXCL1, PCNA, ABCB1 (MDR1), and IGF1R [51–53,56,77,86,87] (Figure 2). In addition to the activation of target genes, the p53 R248Q/W mutant binds to a number of transcription factors or co-factors, including MRE11, p63, and TopBP1. The cellular effects include a reduction in the response to DNA damage, increased genomic instability, increased invasion, and regulation of cell cycle genes [73]. In addition to p63, the R248 mutant interacts with another p53 homolog, p73 [67]. This interaction of R248 with p73 only occurs in the background of a codon 72 single nucleotide polymorphism (SNP) that encodes an arginine or a proline (P72 or R72). The R72 polymorphism binds with higher affinity to p73 than the P72 variant, thus resulting in impaired p73-mediated gene target activation, thereby inhibiting p73 dependent apoptosis [79]. Short peptides have been designed to interfere with oncomorphic p53 binding to p73 [66,88]. Consistent with a role for *R248* mutations in chemoresistance, disruption of this complex sensitizes cells to adriamycin and cisplatin and induces apoptosis. These data also highlight that, while mutation at *R248* is widely considered to predict for resistance to therapy, patients must also be screened for the SNP at codon 72 to achieve an accurate prediction for response.

### 3.3. R175 p53

The p53 oncomorph R175H is the third most common *TP53* mutation in all cancers and is the best characterized mutation. Many different types of cancer cell lines that harbor this mutation as well as murine cancer models have been used to study the oncogenic properties of R175H, and these studies have suggested a role in chemoresistance [40]. Transgenic expression of p53 R175H produces a nearly identical phenotype to the mutation at *R273H*: greater tumorigenic potential with enhanced proliferative capacity [35]. Moreover, transgenic mice expressing R175H p53 in the mammary epithelium increases susceptibility to chemical carcinogenesis with a shorter latency for tumor development as compared to *p53*^−/−^ mice [89,90]. In addition, tumors from *R175H TP53* transgenic mice display increased genomic instability. In experiments in lung cancer cells devoid of p53, overexpression of the oncomorph R175H p53 enhances resistance to both etoposide and cisplatin [40]. Furthermore, our studies indicate that expression of R175H p53 in endometrial cancer cells mediates resistance to paclitaxel in a mechanism that involves maintenance of the G2/M checkpoint [91].

Similar to the R273 and R248 p53 oncomorphs, the R175H mutated protein can interact with proteins NF-Y, p63, p73, and SP-1 to increase cell cycle progression, survival, metastasis, and chemoresistance [57,61,79,81,82]. The increased metastases in some of these animal models may be in part due to the activation of the EGFR/PI3K/AKT pathway [82,92]. These data are consistent with a previous study by our group in which inhibition of EGFR only synergized with chemotherapy in the absence of R175H p53 [91]. In addition to new protein interactions, the R175H mutant is capable of binding DNA and inducing direct transcriptional changes that can create a pro-survival and chemoresistant phenotype. For instance, induction of c-Myc, CXCL1, MAPK family genes, and cyclins A2, B1, and B2 by R175H oncomorph increases proliferation [51–54,57,93]. Another consequence of the R175H oncomorph is the inhibition of genes involved in apoptosis, including NF-κB (p52) and ABCB1 (MDR1) [78,94,95] (Figure 2).

### 3.4. Y220C p53

The *Y220C* mutation occurs in 3.74% of ovarian carcinomas, though it has not been as extensively studied as the other *TP53* mutations. Though it is not defined by others as an oncomorphic mutation, one might speculate it has oncomorphic activity given its reported interaction with p63 and p73 and its transcriptional activation of stathmin, which, as a microtubule destabilizer, is implicated in chemoresistance in many solid tumors [67,96].

In contrast to the lack of understanding of its functional properties, several structural studies have provided novel insight into the *Y220C* mutation. Specifically, the mutated codon is not in direct contact with DNA but rather creates a binding pocket in the core domain of the protein [97] that is a druggable interface. Rational drug design and screening led to the identification of PhiKan 083, a small molecule that is able to bind Y220C p53 in this binding pocket and stabilize the protein [98]. Importantly, stabilization of the mutant protein restores WT p53 conformation and thereby normal function. To date, the effect of this drug has not been explored in *in vivo* cell or mouse models of cancer. Thus, the binding pocket presents an opportunity for a novel strategy of cancer treatment based on the idea of restoring WT p53 function and thereby sensitivity to DNA damaging chemotherapy.

## 4. Methods of Identifying and Understanding Oncomorphisms

**Figure 3:**
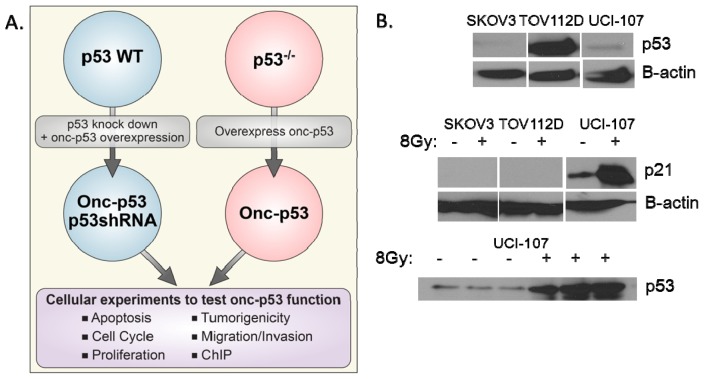
Proposed strategies for uncovering oncomorphic p53 functions: (**a**) Example of an experimental outline used to understand the function of specific *TP53* mutants. Two cellular models are employed: (**1**) In cells containing WT p53, an shRNA can be stably expressed to knock down endogenous p53, while simultaneously overexpressing an shRNA-resistant oncomorphic p53 mutant; (**2**) Cells with *LOF TP53* mutations that do not produce a p53 protein can be used to overexpress oncomorphic p53 variants. Both of these models can then be used to examine the effect of each p53 mutant compared to control cells; (**b**) Example of identifying endogenous p53 function in various ovarian cancer cell lines. Top panel, baseline p53 expression in SKOV3 cells (*LOF TP53*, nonsense mutation), TOV112D (oncomorphic p53, R175H), and UCI-107 cells (WT p53). Middle panel, expression of p21, a marker of WT p53 activation, was assessed 10 h after 8 Gy radiation-induced DNA damage. Bottom panel, stabilization of p53 expression was demonstrated 10 h after irradiation.

Given the vast heterogeneity of tumors, it is reasonable to assume that each oncomorphic *TP53* mutation has context-specific effects. Additionally, each oncomorphic mutation may have differing actions in response to different cancer therapeutics used. Experiments should be performed to compare the various oncomorphic mutants in the same parental cancer cell line in order to reduce variability. Two main strategies should be utilized for these studies: over-expression of oncomorphic mutants in p53-null cells, as well as knockdown of WT p53 concomitant with re-expression of the oncomorphic p53 variants (Figure 3a).

Most of our current knowledge of the oncomorphic functions of *TP53* mutants has been elucidated by over-expressing mutants in p53-null cell lines. Even though forced overexpression represents an artificial biological system, ovarian cancer tumors have high levels of stable p53 mutants, thus justifying this method. While it is advantageous to examine oncomorphic p53 mutants in cells with an intact p53 response pathway, biologic effects could be due to dominant negative activity of the mutant p53 rather than oncomorphic activity. To avoid dominant negative interferences, knockdown of WT p53 using siRNA-mediated gene silencing combined with overexpression of oncomorphic p53 could examine oncomorphic activity. This distinction is essential and physiological, as most advanced ovarian cancer patients have a loss of heterozygosity at the *TP53* locus [99,100].

After establishing the expression models, the next step is to examine WTp53 and oncomorphic p53 function. Several different aspects of the p53 pathway should be evaluated, namely, cell cycle arrest and apoptosis. The most direct method of testing the normal function of p53 is to induce DNA damage and measure p21^WAF1^ induction (Figure 3b). Other key cellular events that might be examined to establish oncomorphic activity include apoptosis, cell cycle progression, proliferation, migration, invasion, colony formation, cytotoxicity and chemoresistance. Moreover, understanding the novel protein binding partners as well as the transcriptional impact of changes in the DNA consensus binding sites associated with each oncomorphic p53 may shed light on their unique pathological functions.

## 5. Conclusions and Consequences for the Future

The process of carcinogenesis involves gaining oncogenic activities as well as losing tumor suppressive activities. Tumors that acquire an oncomorphic *TP53* mutation get a double hit—as a consequence of a single mutation, tumors lose WT p53 tumor suppressive activity and gain a new oncogenic tumor promoter. Almost every advanced serous ovarian tumor contains a *TP53* mutation. We believe that these mutants cannot be binned into two simplistic categories (mutant *vs.* WT) since clearly all *TP53* mutants are not equal. Ovarian cancer is an ideal model to study p53 mutant biology because mutations in this gene are so common. Based on our calculations, oncomorphic mutations may comprise up to 20% of all mutations in ovarian tumors. However, a single therapy may not be adequate for all tumors with oncomorphic *TP53* mutations. Extensive research *in vitro* as well as *in vivo* in mouse models is necessary to examine the biology of each mutation, particularly those that occur with high frequency. The studies described above will pave the way for appropriate comparisons of specific mutants, and possibly identify common signaling pathways that can be targeted for anti-cancer drugs. For example, exciting new methodologies may help progress the field of mutant p53 research by attempting to find oncomorphic p53 binding consensus sequences, as well as transcriptome sequencing to identify oncomorphic p53-associated biomarkers that can be used as predictive tools for resistance to chemotherapy. It will be further necessary to complement *in vitro* studies with animal models of ovarian cancer to understand the function of these oncomorphs and identify drug strategies that will be most useful.

As new technologies helps advance methodologies used to study mutant p53 biology, the most useful information will be discovering commonalities between the mutants. Understanding the basic science of *TP53* oncomorphic mutations can lead to identifying better therapies for patients with oncomorphic mutations in their tumors, and potentially avoid the high incidence of chemoresistance that is currently one of the barriers to improving survival for this deadly disease.

One alternative approach is the idea of restoring WT p53 function. The concept of restoring WT p53 activity is strongly supported by *in vitro* and *in vivo* studies as well as several clinical trials showing that restoration of WT p53 function causes rapid tumor regression in mice and prolonged survival in humans [101–105]. Various strategies have been employed with the ultimate goal of restoring WT p53 function. The most common method used to date is gene therapy, namely introducing a copy of the WT *TP53* gene into tumors using an adenovirus. Excitingly, 50% (8 of 16) women with recurrent ovarian, peritoneal, or fallopian tube cancer that were treated with the replication-deficient adenovirus encoding human recombinant WT *TP53* (SCH 58500) showed a decrease in serum CA125 levels, indicative of clinical response, with minimal side effects [104]. Moreover, combination of WT *TP53* gene therapy with chemotherapeutic drugs such as cisplatin synergized to enhance clinical efficacy [104]. Unfortunately, an international randomized phase II/III trial of WT *TP53* gene therapy in ovarian cancer was closed after the first interim analysis because adequate therapeutic benefit was not achieved [105]. A limitation of these studies may have been the presence of an oncomorphic p53 protein that could impose a dominant negative effect on the therapeutic WT p53 and impede success.

Other approaches such as targeting cellular proteins responsible for stabilizing mutant p53 are another route that may bring success. A recent development involves inhibiting the heat-shock protein HSP90, which chaperones many mutant p53 proteins [106,107] and prevents their degradation by the E3 ubiquitin ligase MDM2 [108,109]. The interaction between the heat shock protein and its client proteins can be disrupted by acetylation of HSP90, posing an exciting opportunity for the use of HSP90 inhibitors as well as deacetylase inhibitors such as the FDA-approved SAHA [110,111].

In summary, the information gained from studying the mutant p53 transcriptome and interactome described in this review has solidified the foundation for the development of strategies that can one day be used to treat the large number of cancer patients that harbor *TP53* mutations.
